# MicroRNA Gene Networks in Oncogenesis

**DOI:** 10.2174/138920209787581299

**Published:** 2009-03

**Authors:** Alexandra Drakaki, Dimitrios Iliopoulos

**Affiliations:** 1 Caritas St Elizabeth Medical Center, Tufts University, Boston, MA, USA; 2 Department of Biological Chemistry and Molecular Pharmacology, School of Medicine, Harvard University, Boston, MA, USA

**Keywords:** microRNAs, gene regulation, gene networks, carcinogenesis.

## Abstract

MicroRNAs are small non-coding RNAs that regulate gene expression at the transcriptional or posttranscriptional level. They are involved in cellular development, differentiation, proliferation and apoptosis and play a significant role in cancer. Examination of tumor-specific microRNA expression profiles has revealed widespread deregulation of these molecules in diverse cancers. Several studies have shown that microRNAs function either as tumor suppressor genes or oncogenes, whose loss or overexpression respectively has diagnostic and prognostic significance. It seems that microRNAs act as major regulators of gene expression. In this review, we discuss microRNAs’ role in cancer and how microRNAs exert their functions through regulation of their gene targets. Bioinformatic analysis of putative miRNA binding sites has indicated several novel potential gene targets involved in apoptosis, angiogenesis and metastatic mechanisms. Matching computational prediction analysis together with microarray data seems the best method for microRNA gene target identification. MicroRNAs together with transcription factors generate a complex combinatorial code regulating gene expression. Thus, manipulation of microRNA-transcription factor gene networks may be provides a novel approach for developing cancer therapies.

## 1. INTRODUCTION

We are in the beginning of a new era in cancer research that started with the identification of a novel category of genes, the microRNAs. They consist a new class of non-protein-coding small RNAs, which regulate the expression of more than 30% of protein-coding genes at the post transcriptional and translational level [1]. Each one can control hundreds of gene targets by translational repression, mRNA cleavage, and mRNA decay initiated by miRNA-guided rapid deadenylation [1, 2]. They regulate cell proliferation and apoptosis and function as oncogenes or tumor suppressors [3, 4]. It is important to mention that so far they were considered to be negative regulators but recent studies showed that can also have positive effect in gene expression [5]. More than 50% of microRNA genes are located in cancer-associated genomic regions or in fragile sites, suggesting that miRNAs may play a more important role in the pathogenesis of a limited range of human cancers than previously thought [6].

The major steps for cancer development include initiation, promotion, malignant conversion, progression, and metastasis [7]. Many factors influence the development of cancers through inhibition or promotion of tumor development. Actually there is a combined interaction of both tumor suppressors and cancer inducers genes, the so-called oncogenes. The unique expression profiles of different microRNAs in different types of cancers and at different stages in one cancer type suggest that microRNAs can function as novel bio-markers for disease diagnostics and may present a new strategy for microRNA gene therapy [8]. Interestingly, more and more evidence indicates that microRNAs also play an important role in many human diseases, ranging widely from cancers, to heart disease, infections as HIV to metabolic diseases [3, 9-11]. This evidence includes, but not limited to, (1) a unique set of miRNAs exists in a specific disease; (2) a unique expression of miRNAs in a certain human disease and (3) aberrant expression of miRNAs in human disease.

## 2. MicroRNA BIOGENESIS

Regarding the history of these novel genes we can briefly mention that the important function of microRNAs was not known until microRNA (let-7) was identified in a variety of organisms such as C. elegans, Drosophila melanogaster and most importantly in humans [12-15]. Since that time, miRNA-related research has become one of the most challenging fields in biomedical science. The first micro RNA (lin-4) was found in 1993 [16] and since then more than 700 microRNAs have been identified and being added in microRNA databases. In order to understand the action of microRNAs we need to know how they are produced. The main characteristic is that they are single-stranded RNAs consisting of 20 to 25 nucleotides. MicroRNAs originate from genes that are called miRNA genes. Specifically, in the nucleus large RNA precursors (whom length vary from several tenths to more than 1000 nucleotides) are named primary microRNAs (pri-miRNAs) and transcribed by RNA polymerase II (Pol II) as well as Pol III with 5’ cap and 3’ poly A tails. These pri-miRNAs are recognized by a microprocessor complex which is composed of the nuclear RNase III Drosha together with its double-stranded RNA binding domain (dsRBD) partner DiGeorge syndrome critical region 8 (DGCR8). The pri-miRNAs are cut into the miRNA precursors (pre-miRNAs) with an approximately 70 nucleotide stem-loop structure [17]. The pre-miRNAs with 2^nd^ hairpin structure are then transported into cytoplasm by the transporter Exportin 5, and this process is RanGTP dependent [18]. In the cytoplasm, the pre-miRNAs were further processed into the 19–24 nucleotide double-stranded miRNA miRNA* complex by another RNase III enzyme, called Dicer, together with its dsRBD partner TRBP [18, 19]. Then, the mature miRNA sequences enter the RNA-induced silence complex (RISC) and target specific gene expression awhile the opposite strand miRNA* sequences are degraded by unknown mechanism.

## 3. THE ROLE OF MicroRNAs IN HUMAN DISEASES

Several studies showed that miRNAs have a fundamental role in a great spectrum of diseases such as cardiovascular, rheumatologic, infectious, inflammatory, autoimmune and metabolic diseases. Poy *et al. *(2004) showed that overexpression of miR-375 inhibited glucose-induced insulin secretion. In conversion, down-regulated miR-375 promoted insulin secretion [20]. It is well known that diabetes is related to insulin resistance. This suggests that miR-375 is a regulator of insulin secretion and may become a novel pharmacological target for the treatment of diabetes. Esau and colleagues (2006) studied the effect of miR-122 in lipid metabolism and observed the decrease in plasma cholesterol level and a significant improvement in liver steatosis by inhibiting miR-122 expression [21]. This was also observed by Krutzfeldt and colleagues [22]. Specifically, a new class of miRNA inhibitors which are known as antagomirs were used to knockdown the miR-122 expression. It was found that down-regulation of miR-122 significantly decreases the plasma cholesterol levels after four days of treatment. This suggests that miR-122 is a key regulator of cholesterol and fatty acid metabolism in the adult liver and a promising therapeutic target for metabolic disease.

Many miRNAs have also identified in several viruses, like HIV, HBV, HCV, Epstein Barr virus (EBV) and human cytomegalovirus (HCMV) [23-27]. Surprisingly, viruses are involved in miRNA production but the exact mechanism for virus-encoded miRNA biogenesis is still unclear. One important function of miRNAs is to control viral replication when the virus infects a cell and in that way to further control virus infection. A liver-specific miRNA, the miR-122 (which was mentioned previously) modulates HCV RNA abundance and HCV replication [28]. In that study it was noticed a significant loss (about 80%) of autonomously replicating hepatitis C viral RNAs by knocking down miR-122.

Regarding the effect of microRNAs in neurologic diseases, they regulate brain and neuron development [29] aberrant expressions of these miRNAs are associated with several neuronal diseases, including Tourette’s syndrome [30], Alzheimer’s disease [31], schizophrenia and schizoaffective disorder [32].

As concerns the cardiovascular diseases, miRNAs play an important role in cardiac development and contractility. It is found that several heart diseases are associated with the aberrant expression of certain miRNAs. For example, cardiac-specific inhibition of miR-1 expression in infarcted rat hearts relieved arrhythmogenesis [33]. These suggest that miR-1 may have an important pathophysiological function in heart failure and may serve as a potential antiarrhythmic target for gene therapy. Furthermore, recent studies showed that another miRNA, miR-133, was associated with cardiac hypertrophy [34]. Overexpression of miR-133 inhibits hypertrophic symptom in both neonatal and adult mouse myocytes whereas, down regulation of miR-133 enhances hypertrophic growth in mouse heart [34]. This suggests that miR-133 is involved to the pathogenesis of heart hypertrophy and over expression of miR-133 in a hypertrophic heart may serve as a novel gene therapy for preventing pathological cardiac hypertrophy.

## 4. MicroRNAs IN CANCER

Cancer is the first leading cause of death after cardiovascular diseases. It has been shown that microRNAs are involved in carcinogenesis development. In order to identify the exact role of microRNAs in cancer pathogenesis, specific microRNAs were overexpressed or knocked down and the initiation and development of different types of malignancies were observed. Recognition of miRNAs that are differentially expressed between tumor tissues and normal tissues may help to identify those miRNAs that are involved in human cancers and further establish the apparent pathogenic role of miRNAs in cancers [35]. When cells exhibit abnormal growth and loss of apoptosis function, it usually results in cancer formation. Cheng *et al.* showed that microRNAs regulate mechanisms such as cell growth and apoptosis [36]. Most of them are related to different types. For example let-7 may control lung cancer development, or at least play a critical role in the pathogenesis of lung cancer. Takamizawa *et al.* observed that the expression levels of let-7 were frequently reduced in both *in vitro* and *in vivo* lung cancer studies; reduced let-7 expression was significantly associated with shortened postoperative survival, independent of disease stage [15]. Regarding breast cancer, Iorio *et al.* found that the miRNA expression patterns were significantly different between normal and neoplastic breast tissues; miR-125b, miR-145, miR-21, and miR-155 were significantly reduced in breast cancer tissues [35]. They also observed that the expression of miRNAs was correlated with specific breast cancer biopathologic features, such as tumor stage, proliferation index, estrogen and progesterone receptor expression, and vascular invasion. Colorectal neoplasia (colon cancer) is also associated with alteration in miRNA expression. Michael *et al.* identified 28 different miRNAs in colonic adenocarcinoma and normal mucosa, and found that the expression of two mature miRNAs, miR-143, and miR-145, was consistently reduced at the adenomatous and cancer stages of colorectal neoplasia [37]. Many factors influence the development of cancers: some inhibit tumor development (tumor suppressors), while others promote cancer development (cancer inducers). The formation of cancer is the combined interaction of both tumor suppressors and cancer inducers. Several experiments suggest that microRNAs may function as a novel class of oncogenes and tumor suppressor genes [38]. Those miRNAs whose expression is increased in tumors may be considered as oncogenes. These oncogene miRNAs are known as oncomirs and promote tumor development by negatively inhibiting tumor suppressor genes and/or genes that control cell differentiation or apoptosis. As opposed to oncomirs, some miRNAs expression is decreased in cancerous cells. These types of miRNAs are considered tumor suppressor genes. Tumor suppressor miRNAs usually prevent tumor development by negatively inhibiting oncogenes and/or genes that control cell differentiation or apoptosis. miRNA let-7 is one of the founding members of the miRNA family and highly conserved [39]. The highest levels of let-7 expression occur in differentiated adult tissues [40]. Inappropriate expression of let-7 results in oncogenic loss of differentiation. In humans, let-7 is located at a chromosome region that is usually deleted in human cancers [6].

## 5. MicroRNAs CANCER SIGNATURES HAVE DIAGNOSTIC AND PROGNOSTIC SIGNIFICANCE

The development of microRNA microarrays have been useful in analyzing the microRNAome in hundreds of clinical samples [41]. Comparison of microRNA expression levels between normal and tumor derived from fresh-frozen or paraffin-embedded tissue samples revealed the microRNAs involved in carcinogenesis. Tumors originated from various tissues can be classified solely on the basis of microRNA expression profiles. The first pilot study performed by Lu *et al.* revealed that 129 microRNAs were able to classify human cancers [42]. Specifically these microRNAs were found down-regulated in cancer relative to normal tissues, suggesting a suppression of microRNA expression during carcinogenesis. In addition, microRNA profiles were informative reflecting the developmental lineage and differentiation state of tumors. Therefore, in a global sense, miRNAs might function to drive cells into a more differentiated state, and the expression profiles of miRNAs in tumors compared with normal tissues might represent the degree of differentiation in those cells.

The advantage of microRNAs relative to mRNA gene signatures is shown by the ability of microRNAs to classify poorly differentiated cancers while mRNA gene signatures failed to classify them. A very recent study by Rosenfeld *et al.* suggested that microRNAs can accurately identify cancer tissue origin [43]. This is of great clinical importance because microRNAs may be used identifying the tissue in which cancers of unknown primary origin arose. In this study using microRNA microarray data from 253 samples, they were able to classify 22 different cancer types using a 48 miRNA classifier with accuracy >90%. All these findings reveal the potential of microRNA profiling in cancer diagnosis.

Furthermore, microRNAs except distinguishing normal and cancers have been correlated with survival in cancer patients. Recently Yanaihara *et al.* identified statistical unique profiles, which could discriminate lung cancers from noncancerous lung tissues as well as molecular signatures that differ in tumor histology [44]. MicroRNA expression profiles correlated with survival of lung adenocarcinomas, including those classified as disease stage I. High miR-155 and low let-7a expression correlated with poor survival by univariate analysis as well as multivariate analysis for hsa-mir-155. Another study from the same group revealed that high expression of miR-21 is associated with poor survival and poor therapeutical outcome in colon adenocarcinomas [45]. Recently Bloomston M *et al.* showed that microRNA expression patterns were able to differentiate pancreatic adenocarcinomas from normal pancreas and chronic pancreatitis [46]. miRNA expression profiles can also be used to distinguish two subtypes of diffuse large B cell lymphoma (DLBCL): germinal center B cell-like (GCB) and activated B cell-like (ABC) DLBCL, in which miR-21, miR-155 and miR-221 were more highly expressed in ABC-type than GCB-type DLBCL [47]. Those suggest that miRNA expression profile is a feasible novel biomarker for early detection of cancers, particularly for poorly understand tumors.

## 6. MicroRNA’s GENE TARGETS

Above we described how microRNAs can be used as prognostic or diagnostic markers in cancer and predict therapeutic outcome. However in order to understand in greater detail microRNAs role in carcinogenesis we should delineate their mechanism of action. microRNAs exert their functions through binding in the 3’ UTR of genes and regulation of their expression. MicroRNAs can bind with perfect or imperfect complementarity to each strand of the double stranded RNA and depending on that, regulate gene expression. There are three major known mechanisms for miRNA-mediated gene regulation: translation repression, direct mRNA degradation and miRNA-mediated mRNA decay [2]. Which mechanism controls gene expression is entirely dependent on the degree of miRNA complementarity to their targeted mRNAs. miRNAs bind, in most cases, with imperfect complementarity to their targeted mRNAs and guide mRNA translation repression. However, there are also several miRNAs which directly degrade their targeted mRNAs. The exact mechanism for miRNA-mediated translation repression is still unknown but most likely miRNA-RISC complex inhibit the initiation and/or elongation of protein translation by interacting with a various translation factors [48]. Furthermore, it is shown that miRNAs mediate gene expression by guiding mRNA decay through deadenylation and de-capping process of targeted mRNAs [49], which is completely different from normal translation repression and/or direct mRNA degradation. It is well known that the 3’ poly(A) tail and 5’ cap are very important for mRNA stability and avoiding mRNA decay. When miRNAs guide the removal of the 3’ poly(A) tail and 5’ cap of the targeted mRNAs, these targeted mRNAs will be degraded by cellular enzymes. In a majority of cases, miRNAs bind to their targeted mRNAs at the 3’ UTR with multiple sites. However, miRNAs targeted to the 5’ UTR and/or the open reading frame (ORF) can also repress gene expression [50]. miRNAs interact with their targeted mRNAs primary through the six to eight nucleotides at the 5’end of miRNAs, which is perfectly bound to the targeted mRNAs. This region is called ‘seed’ sequence in miRNAs and is high conserved in a same miRNA family from species to species [51].

The majority (61%) of miRNA genes are located at an intronic region of protein-coding genes; however can also be in regions of exons or intergenes. Interestingly, more than 50% of miRNA genes can be found in cancer- associated genomic regions or in fragile sites, suggesting that miRNAs play an important role in the pathogenesis of neoplasias [6]. They are also located in minimal regions of heterozygosity loss, minimal regions of amplification, or common breakpoint regions that are genetically altered in human cancers.

## 7. BIOINFORMATIC TOOLS FOR MicroRNA TARGET IDENTIFICATION

In the last few years several bioinformatic tools have been developed to predict microRNA gene targets. These programs rely on a combination of specific-base pairing rules and conservational analysis to score possible 3′-UTR recognition sites and enumerate putative gene targets. Predictions based solely on base-pairing rules have as a result a large number of false-positive hits. The number of false-positive hits, as estimated by random shuffling of miRNA sequences, can be greatly reduced however by limiting hits to only those conserved in other organisms [52]. Important parameters to increase the validity of microRNA gene targets are the following: 1) share a high level of sequence complimentarity with positions 2–8 (called seed sequence) of the 5′ side of the miRNA. 2) share a low level of sequence complimentarity in the 5′ side of the miRNA in addition to a high degree of similarity in the 3′ side [53]. It is thought that a minimum 4 nucleotides on the 5′ side must match perfectly and that G:U wobble pairs, despite their energetic stability, serve to disrupt miRNA targeting [54].

Increasing experimental information regarding microRNAs’ gene targets and signaling pathways has helped to improve substantially the microRNA gene target prediction algorithms. Three of these algorithms are been used extensively by most researchers. These are PicTar [55], TargetScanS [56], and miRanda [52]. PicTar is based on perfect seed binding of 7 nucleotides in the 5′ end starting at either the first or second position. The free energy of miRNA:target binding is then computed for seeds with imperfect matches. To delineate a list of predicted target sites, energy thresholds are imposed and then a maximum likelihood score is computed based on conservation across multiple organisms. TargetScanS computes seed binding sites based on perfect complementarity of a 7 nucleotide region conserved across five organisms (human, chimpanzee, dog, mouse and chicken) between bases 2–8 on the 5′ end of the miRNA, while miRanda uses a modified approach to find miRNA binding sites which do not require perfect seed binding. Kiriakidou *et al.* also used a similar approach named “DIANA-microT” to predict miRNA-recognition elements, but used modified initial base-pairing rules that focus on the sizes of allowable bulges in initial seeds [57]. While conservation has been a primary aspect used to filter hits in most target prediction algorithms, it is important to realize that not all target sites are necessarily conserved. Programs such as the newly developed microTar, which relies on base pairing rules and binding free energy calculations, have moved away from reliance on conservation [58].

As the available bioinformatic algorithms predict hundreds of microRNA-gene target pairs, it is evident that experimental data are needed for verification of these pairs. However, as it is known that several microRNAs target gene expression only at the protein and not at the mRNA level, the integration of microRNA along with protein data sets could be considered more effective for microRNA-gene target verification. Recently Huang *et al.* used microRNA with cDNA expression profiling data to identify human microRNA targets using Bayesian data analysis algorithm [59]. Specifically they identifed a network of 1,597 high-confidence target predictions for 104 human miRNAs, which was supported by RNA expression data across 88 tissues and cell types, sequence complementarity and comparative genomics data. The current most commonly used approach for experimentally validating miRNA targets is to use a luciferase reporter construct by cloning the predicted binding site sequence of the miRNA into the 3′-UTR region [60]. However, this approach is not high-throughput and the miRNA binding sequence is taken out of the context of the target transcript. Because miRNAs can block translation by either direct binding at the 3′-UTR or degrading target mRNAs, conventional microarray based approaches only identify portions of the miRNA targets [61].

## 8. MicroRNA GENE NETWORKS

Transcription factors (TFs) are important components of gene regulatory networks. Like TFs, miRNAs are trans-acting factors that interact with many cis-regulatory elements. Therefore it is not surprising that they generate a complex combinatorial code [62]. Moreover, as transcription factors and genes containing binding sites for other transcription factors have a high probability of being targeted by miRNAs. This interplay between microRNAs and transcription factors renders miRNAs key components of gene regulatory networks.

MicroRNAs have several common features with transcription factors. Specifically individual microRNAs and TFs can regulate dozens or hundreds of genes. Furthermore, genes are regulated not by a single, but by a combination of trans-acting factors. Many TFs bind cooperatively to their cognate DNA sequences and recruit other cofactors. Binding site accessibility provides an additional layer of gene regulatory control. *In vivo* occupancy of TF binding sites depends on nucleosome coverage of the site, with nucleosome positioning and remodeling being regulated processes [63]. Similarly, the accessibility of a miRNA recognition site is controlled by RNA binding proteins [64]. Because TFs are subject to miRNA regulation and miRNA cell type specificity, it is not a surprise that miRNAs and TFs are linked to one another in gene regulatory networks.

On the other hand microRNA and TF mechanisms of action have several differences. For transcription to be repressed, a sophisticated machinery needs to be set in place in a subcellular compartment (the nucleus) that is distinct from the production site of the protein product, the cytoplasm. The stability of already transcribed mRNA species sets another limit to the speed with which transcriptional repression can wipe out the expression of a target gene. In contrast, miRNAs can rapidly turn off protein production right at the site of protein production, the ribosome [65]. Furthermore miRNA-mediated control of gene expression is faster due to their small size and noncoding nature. MiRNAs may be produced more rapidly than TFs, thereby decreasing response times to stimuli that induce gene repression. Also, a miRNA-repressed target can also be reactivated more rapidly than a transcriptionally repressed target, because such reactivation may involve the translocation of an already present mRNA to an active ribosome.

Only a few studies have experimentally explored miRNA function in the context of a gene network. An upstream factor (F) could repress the transcription of a target gene (T) and simultaneously activate the transcription of a miRNA (M) that inhibits target gene translation Fig. (**1**). In type I circuits, the transcription rates of both microRNA and target gene are positively or negatively co-regulated by up-stream regulator Fig. (**1a**). On the other hand in type II circuits, the transcription rates of the microRNA and target gene are differentially regulated by the up-stream regulator Fig. (**1b**). In reality exist more complex networks that these previously described. The c-myc/E2F1 and miR-20a circuit, is an example of type I circuit found in cancer Fig. (**1c**). The E2F1 transcription factor is activated by c-myc, which promotes cell cycle progression. Recently, it has been shown that c-myc activates the mir-17-92 cluster [66]. Specifically, miR-20a modulates the translation of E2F1 mRNA *via *binding in its 3′UTR. In addition, endogenous E2F1 directly bind to the promoter of mir-17-92 cluster activating its transcription. This example displays an auto-regulatory feed-back loop between E2F factors and miRNAs from the mir-17-92 cluster. In type II circuits, a miRNA regulates its targets coherently with transcriptional control, thereby reinforcing transcriptional logic at the posttranscriptional level. Type II circuits may be prevalent, as genome-scale studies have shown that predicted target transcripts of several tissue-specific miRNAs tend to be expressed at a lower level in tissues where the miRNAs are expressed [67]. Type II circuit can be found in Drosophila eye development, where Yan is expressed in progenitor cells and miR-7 in photoreceptor cells [68].

## 9. CONCLUDING REMARKS

The microRNA field although it is relatively new, there are more than 3000 publications related to microRNA functions in many different organisms. However there are still many questions remaining unanswered. Do we know all the enzymes and proteins involved in microRNA production and processing? Except seed sequence complementarity, what are other factors contributing to miRNA-target gene recognition? How microRNAs are regulated in the transcriptional level?

On the other hand, we are just starting to uncover the huge potential of miRNAs as novel biomarkers and therapeutic targets for medicine. The small size of microRNAs and their ability to regulate cancer cell growth suggests their therapeutic potential. RNA interference gene therapies have been in use the last few years and most of the strategies used for delivering small interference RNA (siRNA) potentially can be used for microRNA delivery [69]. In the clinic, microRNAs inactivation could be accomplished through delivery of locked-nucleic-acid (LNA) antisense oligonucleotides against microRNAs [70]. These molecules are more stable and less toxic than other cancer treatments in order to target oncogenic microRNAs. The use of conjugated with cholesterol antisense microRNAs seems an effective approach to inhibit microRNA activity according to murine studies [34]. On the other hand, overexpression of tumor suppressor microRNAs could be used to treat specific types of cancer. For example, let-7 family members are found frequently inactivated in several cancer types. Thus let-7 overexpression using viral or liposomal delivery might be useful approaches for microRNA administration. In addition, let-7 overexpression has been shown to restore sensitivity to cytotoxic anti-cancer therapies [71]. Of course there is need to develop novel microRNA delivery methods and treatments that will move microRNA therapies from the laboratory bench to the bedside.

## Figures and Tables

**Fig. (1):**
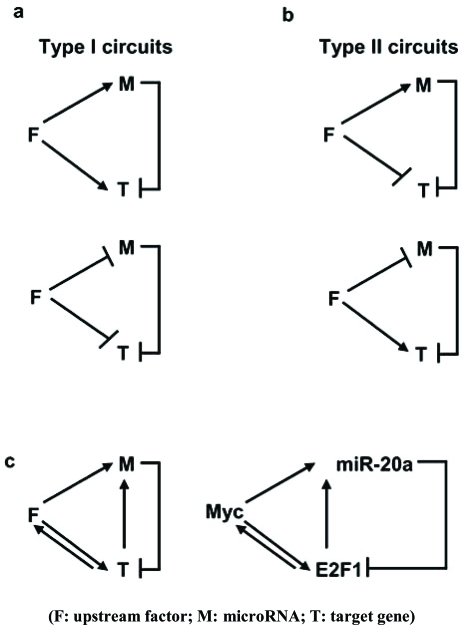
**MicroRNA circuits in cancer. (a)** Type I microRNA-gene circuits. (**b**) Type II microRNA-gene circuits. (**c**) myc/E2F1/miR-20a circuit in cancer.
